# Adverse events of cell therapy clinical trials in human chronic spinal cord injury, a systematic review and meta-analysis

**DOI:** 10.1016/j.reth.2024.03.012

**Published:** 2024-04-22

**Authors:** Esmat Davoudi-Monfared, Reyhaneh Abolghasemi, Fakhri Allahyari, Gholamreza Farzanegan

**Affiliations:** a Health Management Research Center & Department of Community Medicine, Faculty of Medicine, Baqiyatallah University of Medical Sciences, Tehran, Iran; b New Hearing Technologies Research Center, Clinical Sciences Institute, Baqiyatallah University of Medical Sciences, Tehran, Iran; c Neuroscience Research Center, Baqiyatallah University of Medical Sciences, Tehran, Iran; d Trauma Research Center & Department of Neurosurgery, Faculty of Medicine, Baqiyatallah University of Medical Sciences, Tehran, Iran

**Keywords:** Adverse event, Side effect, Safety, Cell therapy, Clinical trials, Spinal cord injury, Review, Meta-Analysis

## Abstract

Spinal cord injury is a lesion with high mortality and significant morbidities. After the primary injury, during six months, a cascade of secondary cellular and molecular events makes the lesion chronic. Recently, cell-based clinical trials as a new procedure have been gradually tested to improve the symptoms of patients. Each treatment method is associated with different adverse events. Based on the PRISMA flow diagram of the identified records, and after multistep screening, finally in 76 reviewed studies with 1633 cases and 189 controls, 64 types of adverse events in 12 categories were recorded in 45 studies. The most common adverse events were transient backache and meningism (90%) and cord malacia (80%). The cell therapy method in which the treatment was associated with more adverse events was Olfactory ensheathing cell and bone marrow mesenchymal stem cell combination therapy in 55%, and the adverse events were less with the embryonic stem cell in 2.33% of patients. In a meta-analysis, the total prevalence of adverse events in cell therapy was 19% and the highest pulled effect size belonged to urinary tract and localized adverse events. Also, the total prevalence of adverse events in 14 cell therapy methods was 18% and four cell types (neural stem cell, bone marrow hematopoietic stem cell, embryonic stem cell, and umbilical cord mesenchymal stem cell) had the most effect. None of the adverse events were reported on the 4 (life-threatening consequences) and 5 (death) grading scales. We concluded that the frequency of life-threatening adverse events following cell therapy clinical trials in chronic spinal cord injury patients is very scarce and can be ignored.

## 1 Introduction

Spinal cord injury (SCI) is a lesion with a high mortality rate and numerous physical, emotional, and social problems for patients. In this injury, the spinal cord, nerve roots, bone structures, and disco-ligamentous components are damaged. Traumatic SCI can be caused by car accidents, falls, violence, and sports [1].

Worldwide, the annual incidence of spinal cord injury in different countries is 15–40 cases per million, and the most common causes are motor vehicle accidents and violence in recreational and work-related activities [2]. Immediately after the initial injury, a cascade of secondary cellular and molecular events occurs, and after six months, the disease enters the chronic phase [3].

Doctors and researchers use different treatment methods to improve chronic SCI patients’ conditions. One of the new methods of treatment is cell therapy, which has received a lot of attention in recent years. Different types of cells from the individual (autologous) or donor (allogeneic) or embryonic cells have been used. Although any new treatment method can have advantages, it is very important to maintain the level of safety and health of patients after using the new treatment procedures. Generally, the most serious side effects of cell transplantation are thrombosis and embolism, tumorigenicity, infection, high fever, and even death [4].

An Adverse Event (AE) is any unfavorable and unintended symptom, sign (including an abnormal laboratory finding), or disease temporally associated with the use of a medical treatment or procedure that may or may not be considered related to the medical treatment or procedure. We used a grading (severity) scale for each AE based on the Common Terminology Criteria for Adverse Events (CTCAE) v5.0 grades with clinical descriptions of severity for each AE based on this general guideline: Grade 1 Mild; asymptomatic or mild symptoms; clinical or diagnostic observations only; intervention not indicated. Grade 2 Moderate; minimal, local, or noninvasive intervention indicated; limiting age-appropriate instrumental activities in daily living (ADL). Grade 3 Severe or medically significant but not immediately life-threatening; hospitalization or prolongation of hospitalization indicated; disabling; limiting self-care ADL. Grade 4 Life-threatening consequences; urgent intervention indicated. Grade 5 Death related to AE [5].

According to the search in electronic medical databases, there was no published systematic review and meta-analysis article about the AE of cell therapy in human chronic spinal cord injury. In this systematic review and meta-analysis, we investigated the safety of cell therapy clinical trials in chronic spinal cord injury.

## 2 Methods

This systematic review was conducted according to the Preferred Reporting Items for Systematic Reviews and Meta-analyses (PRISMA) statement (PROSPERO ID: CRD42021231908). We designed the basic questions for the structure of this research based on the PICO framework:

**P** (participants): Patients with a past history of spinal cord injury at least 6 months after a traumatic event.

**I** (interventions): Human cell clinical trials.

**C** (comparisons): Control group (without cellular intervention), Treated groups (cellular intervention).

**O** (outcomes): Objective and subjective adverse events.

We searched the full text of English clinical trial articles in Cochrane Library, PubMed, SCOPUS, Science Direct, BMJ Journals, ProQuest, Web of Science, and SAGE databases from the database inception to July 20, 2023, at first with sensitive keywords and the Boolean operators *(“cell therapy” AND “clinical trial” AND “human chronic spinal cord injury” AND “safety” OR “side effects” OR “adverse events”*), then with specific keywords and the Boolean operators (*“Mesenchymal stem cell” AND “clinical trial” AND “human chronic spinal cord injury” AND “safety” OR “side effects” OR “adverse events”, “Embryonic stem cell” AND “clinical trial” AND “human chronic spinal cord injury” AND “safety” OR “side effects” OR “adverse events”, “Hematopoietic stem cell” AND “clinical trial” AND “human chronic spinal cord injury” AND “safety OR “side effects” OR “adverse events”, “Olfactory ensheathing cell” AND “clinical trial” AND “human chronic spinal cord injury” AND “safety” OR “side effects” OR “adverse events”, “Schwann Cell” AND “clinical trial” AND “human chronic spinal cord injury” AND “safety” OR “side effects” OR “adverse events”*). Finally, by revisiting the references used in review articles, we found more relevant studies.

For discussion, we downloaded the similar systematic review articles with related keywords and the Boolean operators *(“cell therapy” AND “clinical trial” AND “human chronic spinal cord injury” AND “safety” OR “side effects” OR “adverse events” AND “Systematic review”*). Relevant articles were included in the study and the rest were excluded.

### 2.1 Inclusion criteria

Human cell therapy clinical trials, chronic SCI, English language, full text.

### 2.2 Exclusion criteria

In-vitro studies, animal in-vivo studies, acute and sub-acute SCI, other treatments than cell therapy clinical trials, case reports, duplication, review articles, other languages than English, only abstract, gray literatures (books, news, dissertations, preprints…).

### 2.3 Statistical analysis

We converted the number of adverse events following the cell therapy clinical trials to proportion and percentage (the number of AE per capita) in the case and control groups (p = n/N, n = The number of patients with AE, N = Total number of cases/controls) and Standard error (SE = √pq/N). The form is designed with Excel software (Microsoft Office 2019) and completed based on related data extracted from the articles. The description and regression analysis of data were done employing IBM SPSS statistics (Version 22). We performed a meta-analysis using Stata (Version 17) software. First, we assessed the heterogeneity between the studies by Galbraith's plot. Then we used a random or fixed model and 95% confidence intervals for pooling the percentage of AE in the case of heterogeneous and homogeneous studies, respectively.

## 3 Results

**Fig. 1:**
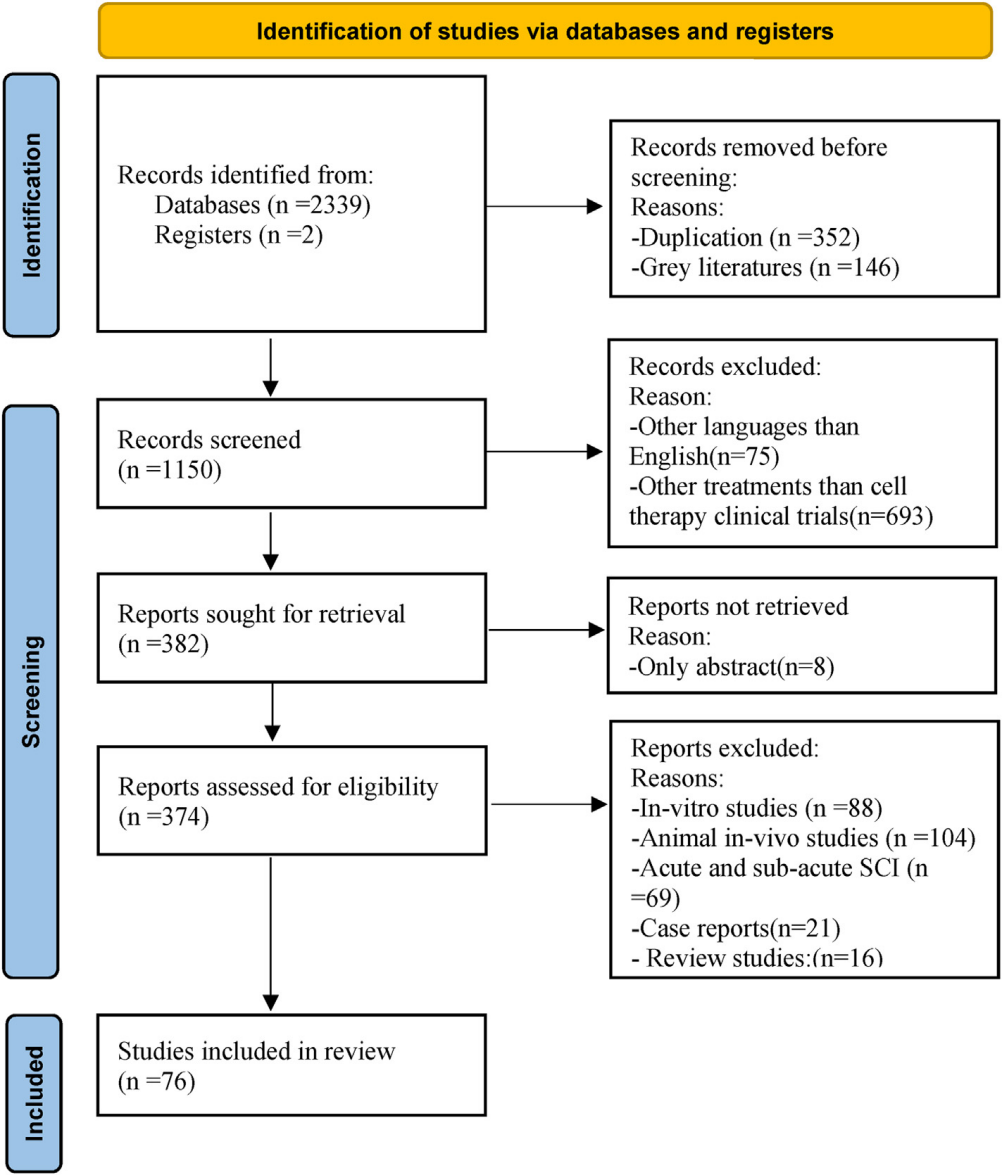
The PRISMA flow diagram records the number of articles found and then makes the selection process transparent by reporting on decisions made at various stages of the systematic review. n: numbers of articles are recorded at the different stages.

The PRISMA flow diagram of the identified records from databases and screening results is shown in Fig. 1. The result of the initial search with keywords in databases and registers was 2341 records. Duplication (n = 352) and gray literature (n = 146) were removed before screening. After primary screening in records, 693 papers were removed because of other treatments than cell therapy clinical trials, and 75 studies because of other languages than English. We didn't find the full text of 8 articles, so they were removed. Eighty-eight in-vitro studies, 104 animal in-vivo studies, 69 acute and sub-acute SCI, and 37 case reports were excluded, and finally, 76 studies were included in the review.

**Fig. 2:**
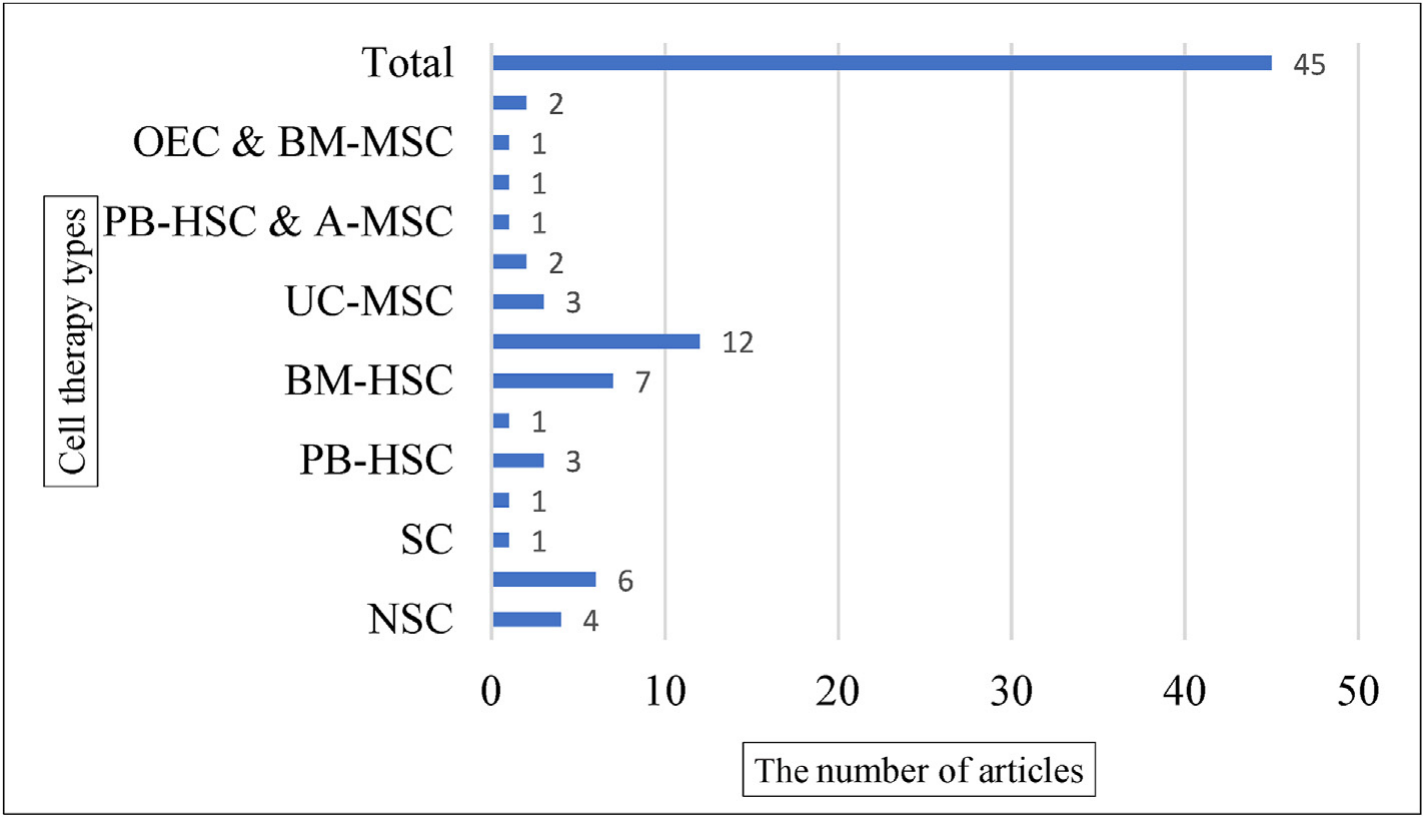
The number of articles that AE of cell therapy was reported.

The greatest number of articles were respectively related to BM-MSC (17 trials), BM-HSC (14 trials), and OEC (14 trials) cells. The average age of all participants was 33.86 years, and most of the participants were male. In 76 reviewed articles with 1633 cases and 189 controls, 64 types of AEs were recorded in 45 studies (Appendix 1). The AEs were reported after cell therapy with BM-MSC in 12 studies [[6], [7], [8], [9], [10], [11], [12], [13], [14], [15], [16], [17]], BM-HSC in 7 studies [[18], [19], [20], [21], [22], [23], [24]], OEC in 6 studies [[25], [26], [27], [28], [29], [30]], NSC in 4 studies [[31], [32], [33], [34]], UC-MSC in 3 studies [[35], [36], [37]], PB-HSC in 3 studies [[38], [39], [40]], A-MSC cells in 2 studies [41,42], and SC& BM-MSC in 2 studies [ [43,44]). In six types of cell therapies, AE was reported only in one study (SC [45], ESC [46], UCB-HSC [47], OEC& BM-MSC [48], OEC& BM-HSC [49], PB-HSC& A-MSC [50]) (Fig. 2).

In the remaining 31 articles, where no AE was reported after cell therapy, the most clinical trials were with OEC (9 studies), then BM-HSC (7 studies), BM-MSC (5 studies), NSC (2 studies), SC (2 studies), SC& BM-MSC (2 studies), PB-HSC (1 study), UCB-HSC (1study), ESC (1study), and UC-MSC (1 study). Since the analysis was not done in these articles, we have not mentioned it in the references.

It should be noted that NSC were of both fetal [33,35] and adult [32,34] origin. But since the cells had fully differentiated neurons, we put them in a separate category of ESC, which are pluripotent undifferentiated stem cells. Umbilical cord mesenchymal stem cell is also extracted from the fetus, but it is different from ESC in nature, so we put it in a different category.

The follow-up period after cell therapy intervention wasn't reported in one study of 45 articles with AE recording [11]. In 44 articles, the average follow-up period was 14.16 months and the minimum time range was one month in two studies [22,40] and the maximum was 51 months in one study [39].

The proportion of AE reported in cell therapy clinical trials is shown in Appendix 1. The most common AEs were transient backache and meningism (p = 0.9) in 90% of patients and cord malacia (p = 0.8) in 80% of patients in case groups. Other AEs had a prevalence below 50%.

We summarized AEs in 12 categories (rows) in 14 cell therapy methods (columns) (Table 1). The most common AEs clusters were urinary tract side effects in 45.6% (p = 0.456 ± 0.0029) and peripheral neural symptoms in 32.9% (p = 0.329 ± 0.015) and the least were vascular lesions in 3.5% (p = 0.035 ± 0.034) of patients. The peripheral neural symptoms and urinary tract AEs were the most common in 8 cell therapy methods. The cell therapy method in which the treatment was associated with more AEs was OEC and BM-MSC combination therapy in 55% of patients (p = 0.55 ± 0.287), and AEs were less with ESC in 2.33% of patients (p = 0.0233 ± 0.01).

**Table 1:** The proportion of AE clusters reported in cell therapy clinical trials.

^∗^ The proportion of AE in one study or the mean of proportions of AE in cell therapies that were more than one article = n/N, n = The number of AE reported in patients, N = Total number of cases.

^∗∗^ The mean of total cell therapies proportions in case groups.

^∗∗∗^ SE (Standard error) = √pq/N. The maximum amount of AE clusters' proportions in each cell type are highlighted.

**Fig. 3:**
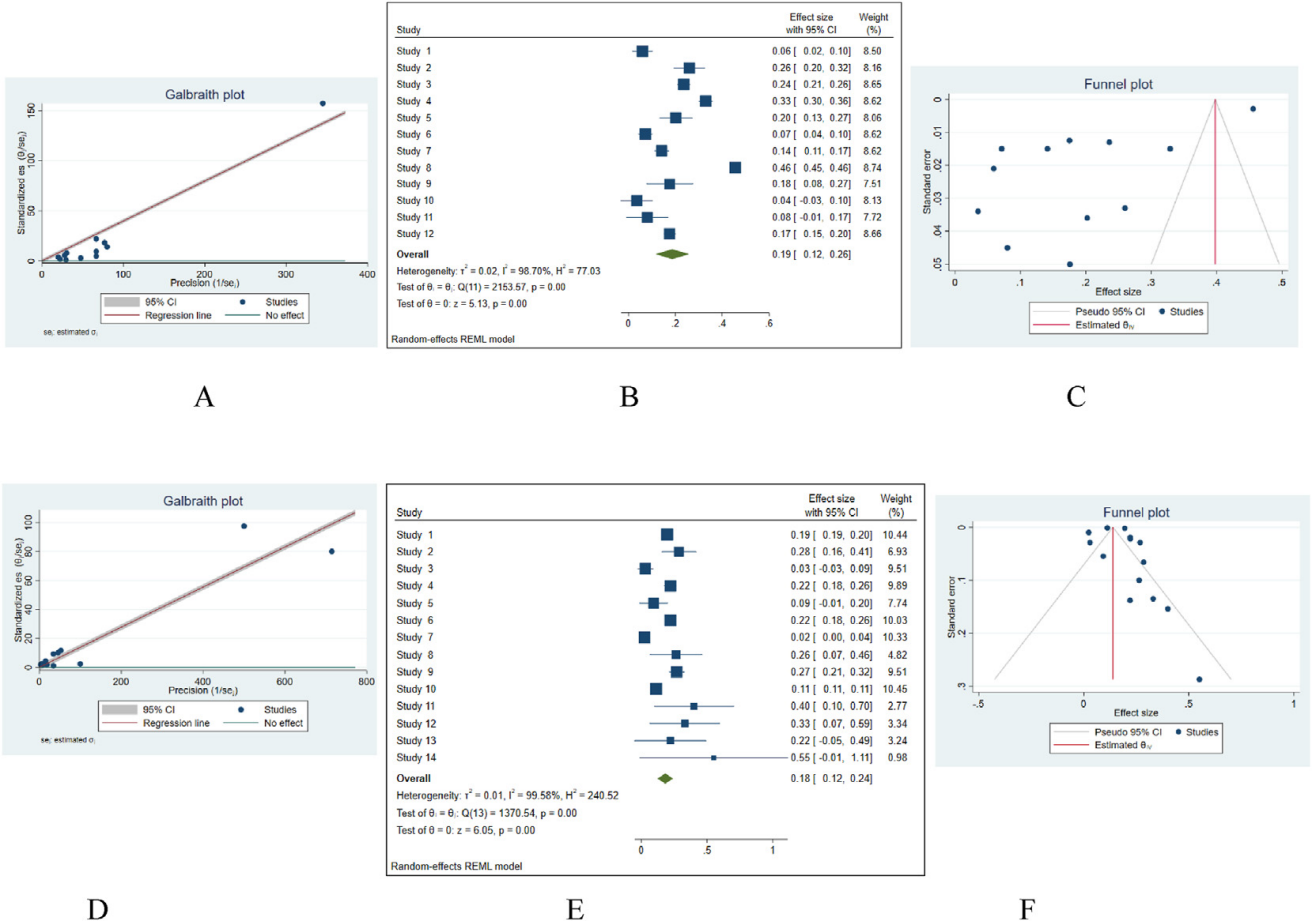
Meta-analysis of AE proportions; ABC: in 12 clusters, DEF: in 14 cell therapy methods. A, D = The Galbraith plot of the heterogeneity test. B, E = The forest plot of Meta-analysis of proportion and standard error. C, F= The funnel plot for publication bias. CI: confidence interval.

We meta-analyzed the mean of AE proportions in case groups. In the Galbraith plot, some studies are outside the range of 95%CI (Fig. 3-A). The result of the meta-analysis with the random-effect model showed a pulled effect size of 0.19 (95%CI = 0.12–0.26). So, in general, we can say that the prevalence of AE in cell therapy is 19%. The effect of 10 clusters of AEs was more than 8% and the respiratory tract AE cluster had the least effect (weight = 7.51%). The heterogeneity is high between studies (I^2^ = 98.70%) (Fig. 3-B, C).

Also, the meta-analysis of the AEs proportions in 14 cell therapy methods showed that the results are very heterogeneous (I^2^ = 99.58%). The pulled effect size with the random-effect model is 0.18 (95%CI = 0.12–0.24). Four cell types (NSC, BM-HSC, ESC, and UC-MSC) had an effect of more than 10%. The effect of PB-HSC & A-MSC was the least (weight = 2.77%) (Fig. 3-D, E, F).

According to linear regression analysis, there is no correlation between the mean of AEs in all types of cell therapies and the mean of follow-up time (Y = 0.133 + 0.005x, ANOVA P value = 0.286).

**Fig. 4:**
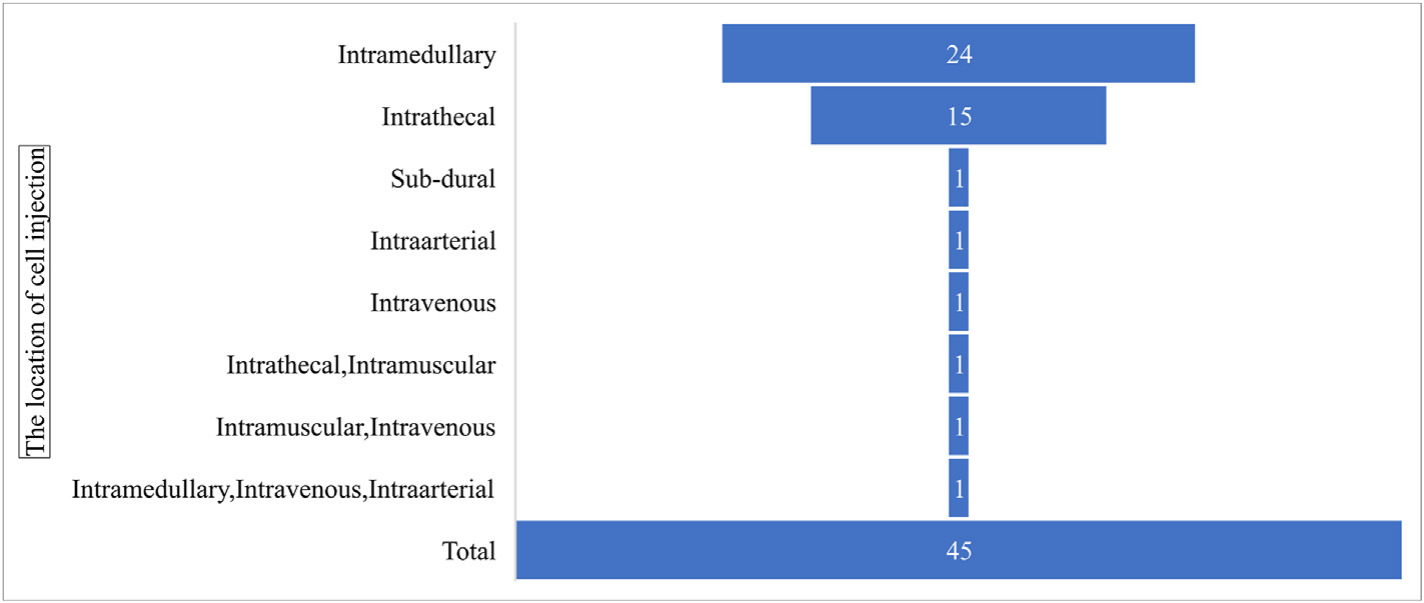
The funnel chart of the number of the articles based on the location of cell injection. Cell therapy is performed in 6 anatomical regions around the lesion (intramedullary, intrathecal, sub-dural, intraarterial, intravenous, and intramuscular). The most injection sites were intramedullary space.

The location of cell injection in different studies is shown in Fig. 4. In general, cell therapy is performed in 6 anatomical regions around the lesion (intramedullary, intrathecal, sub-dural, intraarterial, intravenous, and intramuscular). The most injection sites were intramedullary space (inside the substance or cavity of the cord) and intrathecal space (inside the subarachnoid space) respectively.

Five types of AEs were observed in four studies with BM-MSC in all patients (p = 1). These side effects include the headache [10] (in central nervous system AE cluster), numbness or tingling sensation [12], increase in muscle tension, spasticity, rigidity [7] and pain at the incision site, allodynia, hyperalgesia [9,10] (in peripheral neural symptoms cluster). In these 4 studies, the injection method was intraspinal only in one study [9], but in the other 3 studies, the cells were injected using the intrathecal method (Appendix 1).

In the following, we explain 64 types of AEs in 13 clusters and the severity of AEs in more detail. We meta-analyzed 11 clusters of AEs that had more than one proportion. The vascular lesion was reported only in one research.

### 3.1 Mortality rate

**Fig. 5:**
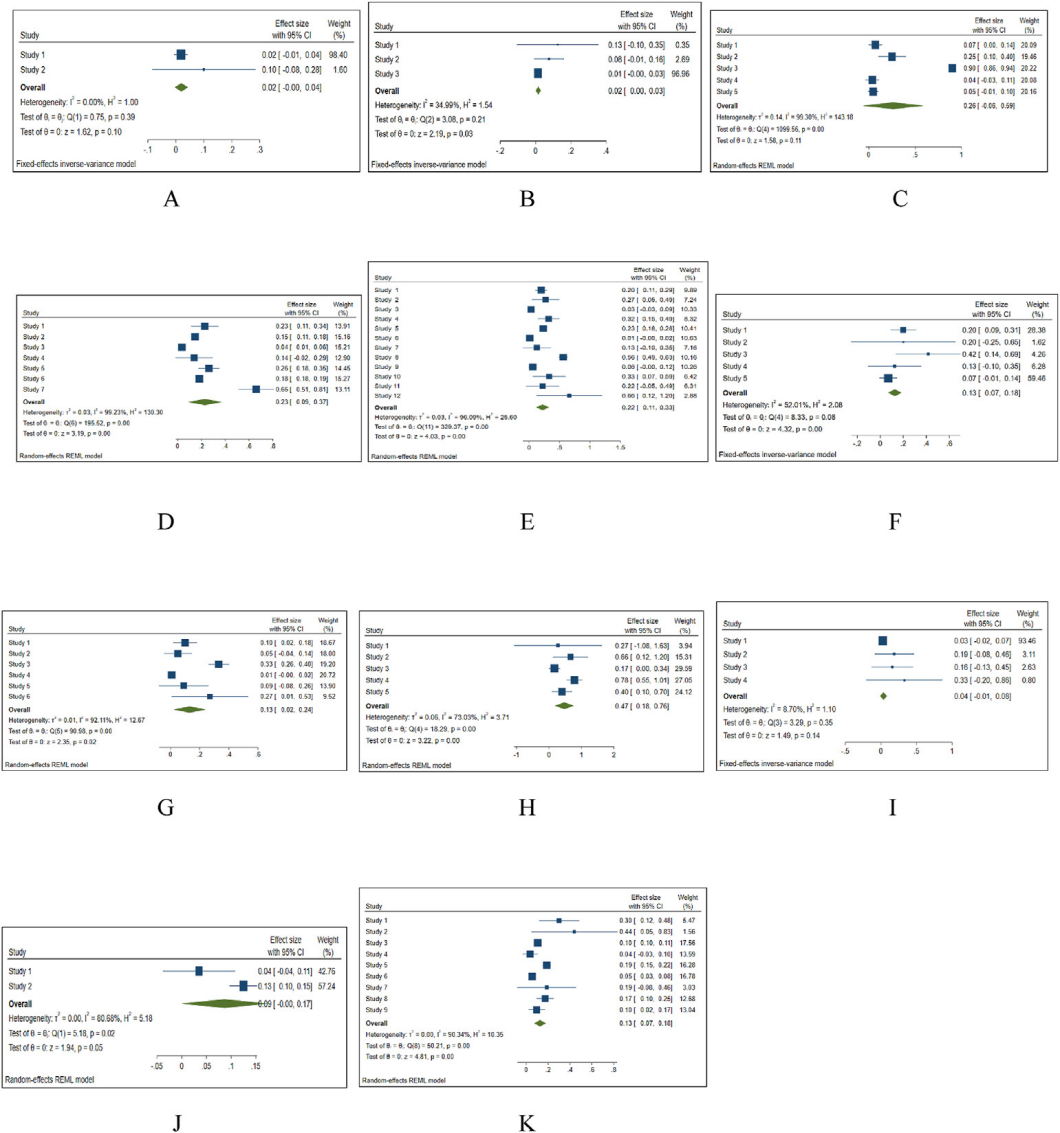
The forest plots of the Meta-analysis of AE proportions. A: Mortality rate, B: Immunological reactions C: Localized AEs, D: Central nervous system AEs, E: Peripheral neural symptoms, F: Skin lesions, G: Gastrointestinal AEs, H: Urinary tract AEs, I: Respiratory tract AEs, J: Endocrine AEs, K: Systemic AEs. CI: confidence interval.

Only in two studies, three deaths were reported in the intervention group. In one study with BM-HSC cells [21], two patients (p = 0.018) died within three months after the intervention due to pulmonary embolism other than the method or cell therapy. In one research with BM-MSC [16], there was one death (p = 0.1) in the treated group before the administration of the study product. In the same study, five patients in the control group (p = 0.5) died due to disease progression. The heterogeneity is very low (Appendix 2) and the result of the meta-analysis with a fixed-effect model caused a pulled effect size of 0.02 (95%CI = −0.00- 0.04) (Fig. 5-A).

### 3.2 The AEs directly related to cell therapy

These AEs include tumor formation, extra-CNS distribution and heterotopic ossification, chondrogenesis foci, and immunological reactions. There were no reports of tumor formation in any study. In a study [47], a participant developed colon adenocarcinoma 21 months after treatment, which the authors cited as possibly unrelated to cell therapy. Extra-CNS distribution and heterotopic ossification and chondrogenesis were not reported in all 76 articles. Various local and systemic allergic AEs occurred in 3 studies (with OEC cells: 1 patient, p = 0.125 [27], PB-HSC cells: 3 patients, p = 0.076 [38], and ESC cells: 3 patients, p = 0.013 [46]). The meta-analysis of these studies results in low heterogeneity (I^2^ = 34.99%) and the pulled effect size with the fixed-effect model is 0.02 (95%CI = 0.00–0.03) (Fig. 5F). The reported immunological reactions included hypersensitivity, itching sensation, and local allergic reaction.

### 3.3 Localized AEs following spinal cord injection

The most localized AE was cerebrospinal fluid (CSF) leak reported in 6 studies total in 9 patients. Meta-analysis of localized AEs according to 5 cell types (NSC, OEC, PB-HSC, UCB-HSC, BM-MSC) showed that the heterogeneity between studies is very high (Appendix 2) and the result of the meta-analysis with random-effect model caused a pulled effect size of 0.26 (95%CI = −0.06–0.59) (Fig. 5B).

### 3.4 Central nervous system AEs

The most common AE in the central nervous system (CNS) was headache. This AE was reported in 20 interventions. The mean occurrence of headache in BM-MSC applications was 0.475, which was the highest number in all types of cells. In the meta-analysis of central nervous system AEs, the heterogeneity is very high (Appendix 2) and the result of the meta-analysis with a random-effect model caused a pulled effect size of 0.23 (95%CI = 0.09–0.37) (Fig. 5C).

### 3.5 Peripheral neural symptoms

The most common AEs in this category were neuropathic pain, increase in muscle tension, spasticity, and rigidity and also pain at the incision site, allodynia, and hyperalgesia. Cell therapy with BM-MSC caused pain at the incision site and allodynia in 4 studies with a mean of 0.725. The heterogeneity between studies is high (Appendix 2). Thus, the meta-analysis with the random-effect model showed a pulled effect size of 0.22 (95%CI = 0.11–0.33) (Fig. 5D).

### 3.6 Skin lesions

The most common skin AE was facial flushing or rash in one study with BM-HSC cells [24] in 5 patients (p = 0.416). In a meta-analysis of skin AEs, the heterogeneity is not important (Appendix 2) and the result of the meta-analysis with a fixed-effect model caused a pulled effect size of 0.13 (95%CI = 0.07–0.18) (Fig. 5E).

### 3.7 Gastrointestinal AEs

The most common AE in the gastrointestinal system was Nausea and vomiting in 5 studies (with BM-HSC cells: 1 patient, p = 0.16 [19,21], ESC: 2 patients, p = 0.008 [46], A-MSC: 1 patient, p = 0.09 [41], and BM-MSC: 3 patients, p = 0.27 [12]). We meta-analyzed 6 cell types that had gastrointestinal AEs. The heterogeneity is high (Appendix 2) and the pulled effect size with a random-effect model is 0.13 (95%CI = 0.02–0.24) (Fig. 5G).

### 3.8 Urinary tract AEs

Urinary tract infection and urinary incontinence were the most AEs in urinary tract system following cell therapy in 7 trials (with NSC: 7 patients, p = 0.58 [33], NSC: 7 patients, p = 0.24 [34], OEC: 2 patients, p = 0.66 [25], A-MSC: 1 patient, p = 0.09 [41], A-MSC: 2 patients, p = 0.25 [42], BM-MSC: p = 0.78 [13], A MSC + PB-HSC: 4 patients, p = 0.4 [50], and NSC: p = 0.083 in control group [31]). The meta-analysis of 6 cell types results in high heterogeneity (Appendix 2) and the pulled effect size with a random-effect model is 0.47 (95%CI = 0.18–0.76) (Fig. 5H).

### 3.9 Respiratory tract AEs

The most common AE in the respiratory tract system was increased sputum and upper respiratory infection in 3 studies (with A-MSC: 2 patients, p = 0.25 [42], BM-MSC: 1 patient, p = 0.16 [14], and OEC + BM MSC: 1 patient, p = 0.33 [48]). In a meta-analysis of respiratory AEs, the heterogeneity is low (Appendix 2) and the result of the meta-analysis with a fixed-effect model caused a pulled effect size of 0.04 (95%CI = −0.01–0.08) (Fig. 5I).

### 3.10 Vascular lesions

The only vascular AE was thrombosis of the vena iliac externa in one study with UC-HSC cells in one patient (p = 0.035) [47].

### 3.11 Endocrine AEs

Hyperthyroidism or low TSH was established in two studies) with UC-HSC cells: 1 patient, p = 0.035 [47], and A-MSC cells: 1 patient, p = 0.125 [42]). The meta-analysis of these two studies results in high heterogeneity (Appendix 2) and the pulled effect size with the random-effect model is 0.09 (95%CI = − 0.00–0.17) (Fig. 5J).

### 3.12 Systemic AEs

The most common systemic AE was Musculoskeletal pain (general, back, neck, shoulder) in 11 studies. Also, the fever appeared in 10 studies. The meta-analysis of 9 cell types results in high heterogeneity (Appendix 2) and the pulled effect size with a random-effect model is 0.13 (95%CI = 0.07–0.18) (Fig. 5K).

Totally, in all AEs clusters, the highest pulled effect size belongs to urinary tract AEs (Pulled effect size = 0.36 (0.08–0.65)) and then localized AEs (Pulled effect size ± 0.26 (−0.06–0.59)) had a relatively higher prevalence in patients receiving cell therapy (Table 2). In the studies that reported the urinary tract and localized AEs, most injection sites were intramedullary.

**Table 2:** The summary of meta-analysis of AE clusters.

^∗^ The I^2^ statistic describes the percentage of variation across studies that is due to heterogeneity rather than chance. For unimportant (I^2^ = 0–30%) and moderate (I^2^ > 30–60%) heterogeneity we used fixed-effect model and for substantial (I^2^ > 60–90%) and considerable (I^2^ > 90–100%) heterogeneity we used random-effect model in meta-analysis.

^∗∗^ Fixed-effect model.

^∗∗∗^ Random-effect model. The highest value of pulled effect size is highlighted.

### 3.13 The grades of AEs

**Fig. 6:**
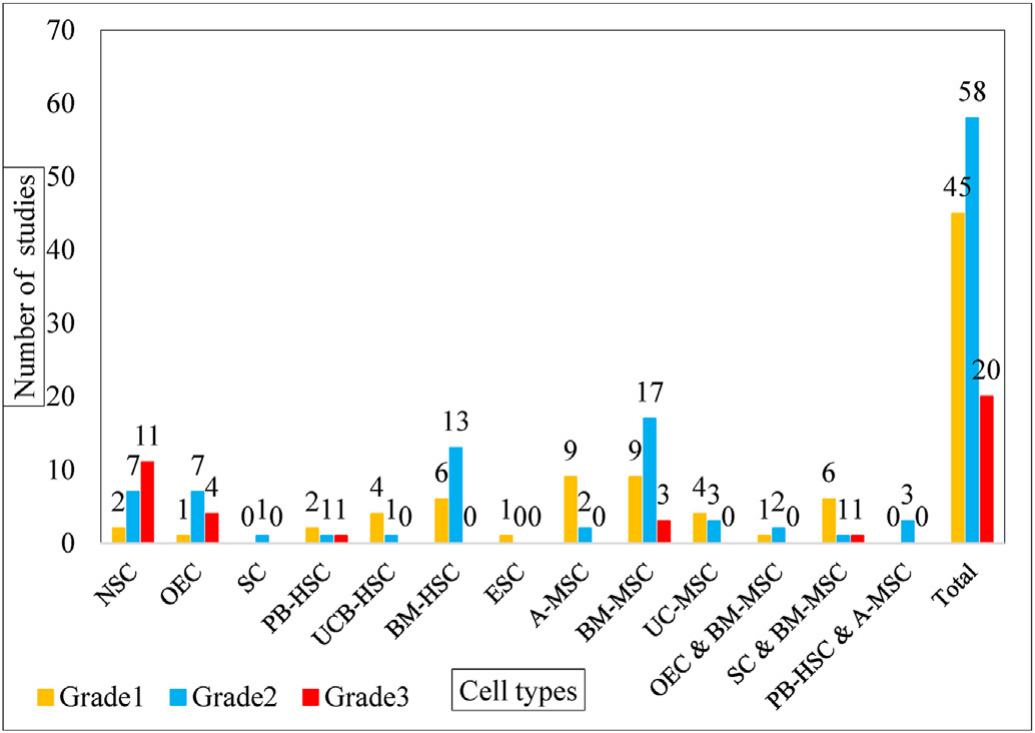
The bar chart of the number of studies that reported AEs based on different cell types. (In all articles, more than one AE is reported).

Grade refers to the severity of the AE. Based on the CTCAE [5] we graded the AEs. None of the adverse events were reported on the 4 (life-threatening consequences) and 5 (death) grading scales (Fig. 6). Seventeen severe AEs (hospitalizations, grade 3) have occurred in 20 studies in which 5 types of cells were used (Table 3). The NSC therapy showed the highest number of severe AE. On the other hand, 3 severe AEs (CSF leak, wound infection, and constipation) occurred in two studies. Other severe AEs were reported in one study.

**Table 3:** The Severe (grade3) AE reported in different cell therapies.

^∗^ The studies that reported the AE in case group.

^$^ The studies that reported the AE in control group.

^#^ In case groups.

Grade 2 (moderate) AEs occurred in 58 studies (in some studies, more than one AE occurred) (Table 4). The most reported moderate AEs were from bone marrow derived cells (BM-MSC:17 reports and BM-HSC:13 reports). The most common AE with moderate severity were neuropathic pain and headache. All reported immunological reactions were moderate (grade 2) severity.

**Table 4:** The moderate (grade2) AE reported in different cell therapies.

^∗^ The studies that reported the AE in case group.

Grade 1 (mild) AEs were reported in 45 studies (in some studies, more than one AE occurred) (Table 5). The use of mesenchymal stem cells (from adipose and bone marrow, each in 9 reports) was associated with the highest prevalence of AEs with mild severity. In terms of the type of AEs, headache was the most common mild AE.

**Table 5:** The mild (grade1) AE reported in different cell therapies.

^∗^ The studies that reported the AE in case group.

## 4 Discussion

### 4.1 Summary of evidence

The safety of cell therapy in chronic SCI treatment is very important. This study analyzed the AE of 14 types of cell therapy clinical trials in human chronic spinal cord injury and showed that in 45 studies of 76 reviewed articles, 64 types of AEs in 12 categories were reported. The most injection sites for cell therapy were intramedullary and intrathecal space. The time range of follow-up period was 1–51 months (mean = 14.16 months). The most common AEs were transient backache and meningism in 90% of patients and cord malacia in 80% of patients in case groups. The most common AEs clusters were urinary tract side effects in 45.6% and peripheral neural symptoms in 32.9% and the least were vascular lesions in 3.5% of patients. The cell therapy method in which the treatment was associated with more AEs was OEC and BM-MSC combination therapy in 55% of patients, and AEs were reduced with ESC in 2.33% of patients.

None of the adverse events were reported on the 4 and 5 grading scales. Seventeen severe AEs (hospitalizations, grade 3***)*** have occurred in 20 studies. The NSC therapy showed the highest number of severe AE. Grade 2 (moderate) AEs occurred in 58 studies. The most reported moderate AEs were from bone marrow derived cells (BM-MSC and BM-HSC). The most common AE with moderate severity were neuropathic pain and headache. Grade 1 (mild) AEs were reported in 45 studies. The adipose and bone marrow derived mesenchymal stem cells were associated with the highest prevalence of AEs with mild severity. In terms of the type of AEs, headache was the most common mild AE.

The noteworthy point is that majority of the AEs reported in the articles are caused by the surgical process and only immunological reactions are directly related to the cell therapy. Also, no cases of death due to the method or cell therapy were reported.

### 4.2 Comparing with previously published reviews

We have compared the results of this study with the results of other review studies. In an overview article, Suzuki H and Sakai T, on preclinical and clinical studies using stem cells for the treatment of chronic SCI, concluded that cell transplantation of NSCs, MSCs, and Schwann cells harvested from various tissues, including iPSC, can be considered as a useful treatment for chronic SCI patients in the near future. They did not report any side effects in their review [51]. In a scoping review by Willison et al., complications of cell therapy in acute to chronic spinal lesions were evaluated. No serious complications were reported from injection through open surgery, lumbar puncture, arterial infusion, or intravenous infusion [52].

The AEs in other review studies are listed according to their severity.

Based on our results, the most common moderate AEs were neuropathic pain and headache following bone marrow derived MSC and HSC cell therapy. Also, the headache was the most common mild AE due to cell therapy with adipose and bone marrow derived MSC. Common mild or moderate AEs in Chen Wc. et al. review of 13 studies included fever, headache, back pain, and numbness [53]. Tang, Q. R. et al. mentioned the pain, fever, and headache as the most common mild and moderate AEs in stem cell transplantation for treatment of spinal cord injury [54]. The most reported AEs were febrile reaction, headache, and neurologic pain with mild or moderate severity in ten studies in Liu, S. et al. systematic review and network meta-analysis [55]. In de Araújo, L.T. et al., study, the most common side events were moderate headache and pain at the site of the lesion [56]. In Kvistad CE. et al., review, fever and headache were the most common AEs, irrespective of administration mode [57]. Analysis of AEs in twelve studies with 502 patients receiving mesenchymal stem cell transplantation showed that the commonly reported mild AEs included fever, headache, and neuropathic pain [58].

The CSF leak, wound infection, and constipation were the more common severe AEs in our review. The CSF leakage has been more common severe AE in other studies [56,59,60]. The wound infection with Staph. Epidermis was reported in Van den Bos, J. et al. systematic review with meta-analysis, in 19 studies [60]. The other severe AE with less prevalence included autonomic dysreflexia, meningitis [59], and pseudo meningocele [59,60]. On the other hand, in the systematic review and meta-analysis by Tang, Q-R. et al., there was no reporting of wound infection, cerebrospinal fluid leakage, intracranial infection, or spinal cord diameter increasing. Suture fracture on the second day after the surgery was reported in one patient [54].

In our review, the NSC therapy showed the highest number of severe AE. In other review articles, prevalence of AE was not analyzed according to the type of cells received.

There were no reports of life-threatening consequences (grade 4) and death (grade 5) associated with cell therapy in the articles we reviewed. In Xu, X. et al. review, three perioperative deaths were reported in two articles: 2 patients who received OECs (Huang et al., 2009) due to hypertensive intracerebral hemorrhage and pulmonary infection, and one patient received macrophages (Lammertse et al., 2012). The authors explained that the cause of death may have been related to obesity [59]. The Huang et al. article was excluded from our review because it was in Chinese. The Lammertse et al. study was conducted in an acute phase. Therefore, it was not among our review articles. In the safety analysis of mesenchymal stem cell treatment in 479 traumatic SCI patients by Kvistad CE. et al., one serious AE was reported and this was a patient who died due to complications after surgery (Li ZY. et al., 2008) [57]. The Li ZY. et al. article could not be downloaded on the internet, so it was not included in our review.

In addition to our article, mortality was not reported in other review articles [55,58]. In these studies, immune reactions were not reported, but in our study, the local and systemic allergic AEs occurred in 3 studies.

It is important to note that in all review articles that have been published so far, similar to our results, the occurrence of AEs directly related to cell therapy such as tumor formation and abnormal tissue proliferation has been rejected [[53], [54], [55],^58^], even up to 6 years after administration [60].

Chen et al. claimed that intrathecal injection was the best transplantation route [53]. Also, in our study, the intrathecal method was associated with fewer AEs than the intramedullary site.

Xu, X. et al. reviewed 44 articles, involving 1266 patients with 6 types of cell therapies: OECs, NSC, MSCs, Schwann cells, macrophages, and combinations of MSCs and Schwann cells. AEs occurred 1144 times in total and the number of AE per capita was less than one case for both the OEC and MSC. The total prevalence of severe AE for all types of transplanted cells was 46.81% [59]. But in our review, the cell therapy method in which the treatment was associated with more AEs was OEC and BM-MSC combination therapy in 55% of patients, and AEs were fewer with ESC in 2.33% of patients.

The results of other review articles similar to our results showed that the frequency of severe AEs after cell therapy in chronic SCI is very low. Therefore, in these patients, where the existing treatments often do not help much in improving their symptoms, cell therapy is promising for the future.

### 4.3 Strengths and limitations

In this review, we encountered some limitations. One of the limitations is the non-uniformity of the AEs measurements in different studies. It is necessary to conduct standardization for clinical trial studies in the recording of AEs. We only reviewed clinical trial articles. This seems inadequate. The clinical trials typically assess only a few patients and thus the chances of detecting rare AEs are small. The clinical trials are usually of short duration and thus cannot identify delayed AE. Therefore, the assessment of safety should use various methods, including spontaneous reporting schemes, post-marketing surveillance studies, and epidemiological investigations.

The transplant route may be an important prognostic factor. However, due to the limitations of the included articles, including the use of several injection methods without mentioning the connection between the injection method and AEs, we failed to analyze the differences in safety between different transplantation routes.

The maximum follow-up time in the clinical trial studies that have been conducted so far was 51 months. This time may not have been enough for the occurrence of some AEs such as tumorigenesis.

One of the important limitations of our study was the exclusion of 75 non-English articles in the first stage of screening of the records. Most of them were Chinese. Some of them might report important results related to our review.

Our review article has an edge over similar articles. In terms of the number of articles for review and the number of cases and controls, our results are more reliable. Also, no review articles have been written specifically about chronic lesions.

## 5 Conclusions

The results of this systematic review and meta-analysis showed that the most common adverse events were transient backache, meningism, and cord malacia. Also, the frequency of severe AE following cell injection in chronic SCI patients is uncommon. In most studies, mild and moderate AEs have been reported, which spontaneously or with conventional treatments improved. Moreover, it showed that the cell therapy method in which the treatment was associated with more adverse events was OEC and BM-MSC combination therapy, and the adverse events were fewer with ESC. The immunological reactions were in moderate severity and they can be prevented and controlled with immunosuppressive drugs.

The results of our systematic review and meta-analysis emphasized that the safety of various cell therapy methods is remarkable.

## Funding

The authors weren't supported by any foundation for writing the manuscript.

## Consent to participate

Not applicable.

## Consent for publication

Not applicable.

## Availability of data and materials

All data generated during the review is included in this published article.

## Code availability

Not applicable.

## Authors' contributions

Dr. R A is the main manager. She created the main conception and design of the research. Dr. E D M collaborated in data extraction from articles and interpretation of results. Dr. F Aland Dr. G F contributed to the overall approval of the work. All authors read and approved the final manuscript.

## Ethics approval

The project was proposed by the Ethics Committee of the Ministry of Health, Medical Education and Treatment of Iran (Ethical code: IR.BMSU.REC.1401.101). The authors have respected the ownership rights of the articles used for review. The authors avoided plagiarism.

## Declaration of competing interest

The authors have no conflicts of interest to disclose.

## Figures and Tables

**Appendix 1:** The proportion of AE reported in cell therapy clinical trials

Side effects clusters	Cell type AE	NSC	OEC	SC	PB-HSC	UCB-HSC	BM-HSC	ESC	A-MSC	BM-MSC	UC-MSC	PB-HSC & A-MSC	SC & BM-MSC	OEC & BM-HSC	OEC & BM-MSC	mean
**Mortality rate**							0.018^#^			0.1,0.5^$^						0.059^&^
**Immunological reactions**	Hypersensitivity, itching sensation, local allergic reaction		0.125		0.076			0.013								0.023
**Localized AEs following** **spinal** **cord** **injection**	Subdural hematoma					0.035										0.035
	Subarachnoid hemorrhage					0.05										0.05
	Spinal cord edema and swelling		0.05													0.05
	Cord malacia		0.8													0.8
	Pneumocephalus.					0.035										0.035
	Posterior reversible encephalopathy syndrome	0.083, ∗														0.083
	Encephalomyelitis									0.023						0.023
	Pseudomeningocele	0.08, 0.034**(0.057)**^**@**^														0.057
	Meningitis		0.05													0.05
	Transient backache and meningism				0.9											0.9
	Syrinx cavity enlargement		0.2, 0.25**(0.225)**,0.25				0.11									0.225
	Cerebrospinal fluid leak	0.08, 0.06**(0.07)**	0.125, 0.15**(0.1375)**			0.035				0.07						0.078
**Central nervous** **system** **AEs**	Vertigo							0.004								0.004
	Somnolence								0.125							0.125
	Aphasia	∗														
	Seizure	0.16, ∗														0.16
	Dizziness	∗								0.05	0.13					0.09
	Headache	0.42, 0.17**(0.295)**					0.017, 0.25, 0.009, 0.125, 0.25, 0.225 (**0.146**)	0.066	0.18, 0.125 (**0.1525**)	0.05, 0.05, 0.8,1, ∗, ∗, (**0.475**)	0.42, 0.05 (**0.235**)		∗		0.66	0.29
**Peripheral neural symptoms**	Ulnar nerve compression	∗														
	Metacarpal hypoesthesia	0.034														0.034
	Paresis		0.33													0.033
	Paresthesia	0.034														0.034
	Autonomic dysreflexia	0.16, 0.1 (**0.13**)														0.13
	Neuropathic pain	0.25	0.2	0.03		0.32	0.33, 0.07		0.125	0.55, 0.3 (**0.425**), ∗, 0.2	0.1		0.25,0.25 (**0.25)**	0.22	0.66	0.265
	Deterioration in neurological level						0.08			0.06						0.07
	Numbness and tingling sensation						0.228 0.5 (**0.364**)			0.31,1 (**0.655**)			0.25			0.423
	Increase in muscle tension, spasticity, and rigidity	0.05, 0.25 (**0.15**)					0.033, 0.25 (**0.1415**)		0.125	0.125,1,0.5 (**0.541**)	0.016	∗	0.75, 0.25 (**0.5**)		0.66	0.3
	Pain at the incision site, allodynia, and hyperalgesia	0.58	0.28					0.008		0.1,1,1, 0.8 (**0.725**), ∗			∗			0.398
**Skin lesions**	Wound infection	0.083, 0.068 (**0.0755**)														0.0755
	Furuncle								0.125							0.125
	Wound hemotoma		0.083										∗			0.083
	Erythema	0.16														0.16
	Scratch	0.25														0.25
	Facial flushing or rash						0.416						∗			0.416
	Excessive sweating									0.069						0.069
	Decubitus pressure ulcer	0.33	0.33													0.33
**Gastrointestinal AEs**	Abdominal pain, discomfort	0.16					0.58	0.008		∗						0.249
	Nausea, vomiting						0.16,0.16 (**0.16**)	0.008	0.09	0.27						0.174
	IBS		0.05													0.05
	Constipation	0.083,0.034 (**0.058**)					0.25	0.008								0.105
	Fecaloma	0.083														0.083
**Urinary tract AEs**	Bleeding at the bladder catheter site	0.16														0.16
	UTI, urinary incontinency	0.58,0.24 (**0.41**), 0.083	0.66						0.09,0.25 (**0.17**)	0.78		0.4				0.484
	Increased creatinine	0.25														0.25
**Respiratory tract AEs**	Chest pain, respiratory tightness								0.125							0.125
	Pneumothorax				0.025											0.025
	Increased sputum, upper respiratory infection								0.25	0.16, ∗					0.33	0.246
**Vascular lesions**	Thrombosis of vena iliac externa					0.035										0.035
**Endocrine AEs**	Hyperthyroidism, low TSH					0.035			0.125							0.08
**Systemic AEs**	Sepsis	0.083, ∗														0.083
	Fever		0.33		0.105		0.319	0.101	0.125	0.1,0.18 (**0.14**), ∗, ∗	0.141					0.18
	Anemia		0.66				0.375,0.2 (**0.28**)									0.47
	Hypotension		0.33													0.33
	Hypertension					0.035	0.125			0.069						0.076
	Hyperthermia									∗						
	Musculoskeletal pain (general, back, neck, shoulder)	0.66, ∗	∗				0.017,0.009,0.25 (**0.092**)		0.25	0.27,0.375 (**0.322**), ∗	0.05					0.274
	Weight loss							0.004								0.004
	Increase of transaminases and hypertriglyceridemia						0.125									0.125
	Depression		0.25													
	Fall	0.16														0.16
	Accidental burns									0.16						0.16

#: The proportion of AE in case groups = n/N, n = The number of AE reported in patients, N = Total number of cases, $: The proportion of AE in control groups = n/N, n = The number of AE reported in patients, N = Total number of controls, **@**: The mean of proportions in case groups in any kind of cell therapy, &: The mean of total cell therapies proportions in case groups, ∗: The cell therapy has been associated with AE occurrence, but the number of affected patients didn't report.

**Figure:**
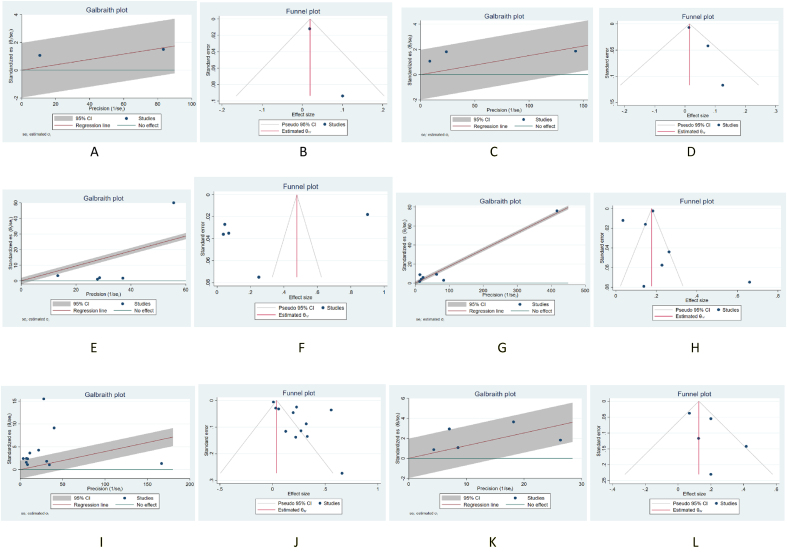

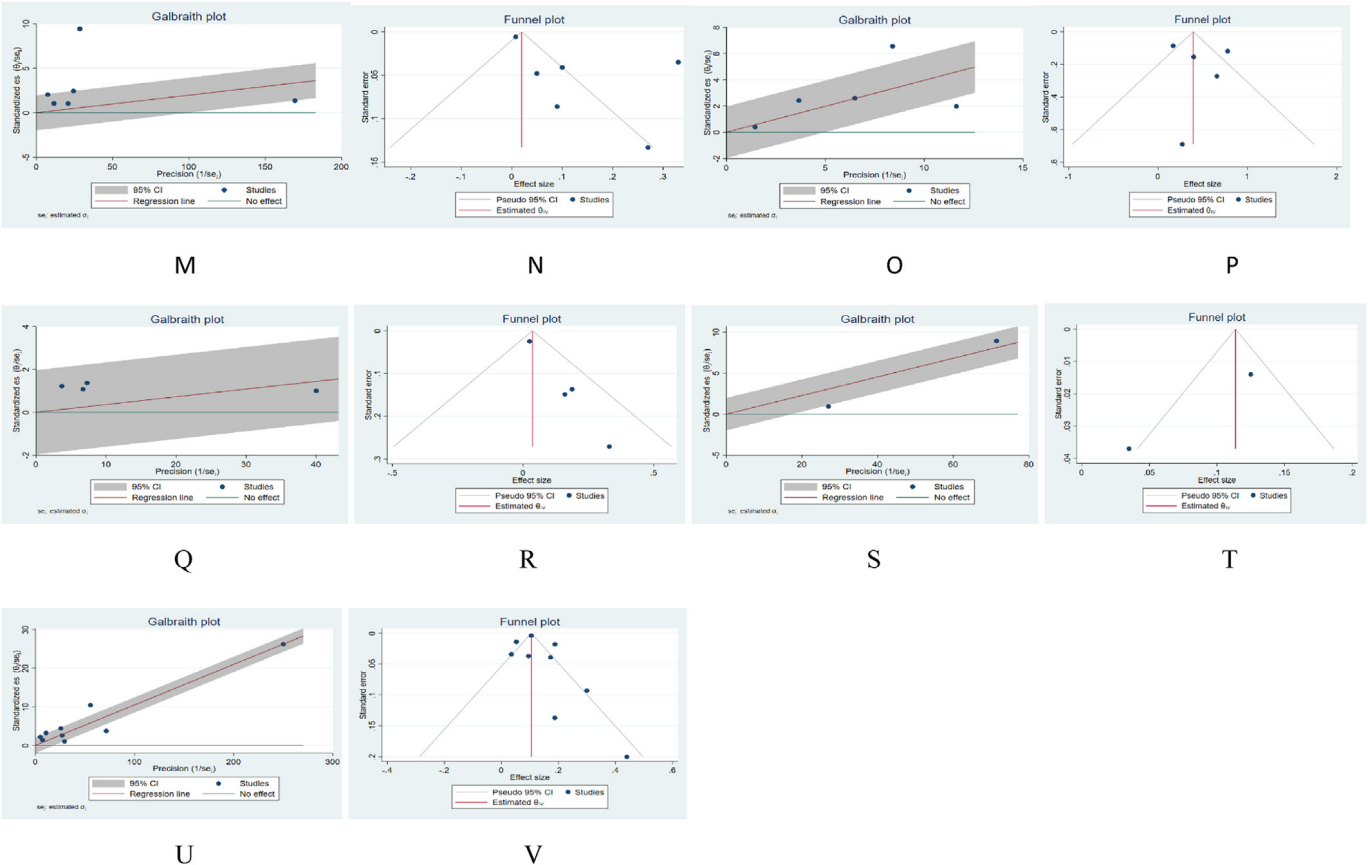
**Appendix 2** Meta-analysis of AE proportions. A, B: Mortality rate, C, D: Immunological reactions, E, F: Localized AEs, G, H: Central nervous system AEs, I, J: Peripheral neural symptoms, K, L: Skin lesions, M, N: Gastrointestinal AEs, O, P: Urinary tract AEs, Q, R: Respiratory tract AEs, S, T: Endocrine AEs, U, V: Systemic AEs, A, C, E, G, I, K, M, O, Q, S, U= The Galbraith plot of the heterogeneity test. B, D, F, H, J, L, N, P, R, T, V= The funnel plot for publication bias. CI: confidence interval .
